# Subclinical Myocardial Fibrosis in Systemic Lupus Erythematosus as Assessed by Pulse-Cancellation Echocardiography: A Pilot Study

**DOI:** 10.3390/jcm11164788

**Published:** 2022-08-16

**Authors:** Alessandro Giollo, Giulia Vinco, Giovanni Cioffi, Francesca Frizzera, Anna Quinternetto, Corinna Bergamini, Marta Dal Porto, Giovanni Orsolini, Margherita Zen, Andrea Doria, Davide Gatti, Flavio Luciano Ribichini, Giovanni Targher, Maurizio Rossini, Ombretta Viapiana

**Affiliations:** 1 Rheumatology Section, Department of Medicine, University of Verona, 37129 Verona, Italy; 2 Rheumatology Section, Department of Medicine, University of Padova, 35128 Padova, Italy; 3 Division of Cardiology, Santa Maria del Carmine Hospital, 38068 Rovereto, Italy; 4 Section of Cardiology, Department of Medicine, University of Verona, 37129 Verona, Italy; 5 Division of Cardiac Rehabilitation, San Pancrazio Hospital, Arco di Trento, 38062 Trento, Italy; 6 Section of Endocrinology, Diabetes and Metabolism, Department of Medicine, University of Verona, 37129 Verona, Italy

**Keywords:** lupus, myocarditis, cardiovascular, glucocorticoids, ultrasound, eSCAR, echocardiography, strain, flares, myocardial

## Abstract

The aim of this study was to examine whether scar imaging echocardiography with ultrasound multi-pulse scheme (eSCAR) can detect subclinical myocardial involvement in systemic lupus erythematosus (SLE). We consecutively recruited SLE patients and controls matched for age, sex, and cardiovascular risk factors. Participants with cardiac symptoms or a prior history of heart disease were excluded. All participants underwent eSCAR and speckle tracking echocardiography (STE) with global longitudinal strain (GLS) assessment. SLE patients were assessed for disease activity and were followed up for 12 months. Myocardial scars by eSCAR were observed in 19% of SLE patients, almost exclusively localized at the inferoseptal myocardial segments, and in none of the controls. GLS was significantly lower in most myocardial segments of SLE patients compared with the controls, especially in the inferoseptal segments. eSCAR-positive SLE patients received a higher cumulative and current dose of prednisone, and had significantly higher levels of anti-dsDNA antibodies (*p* = 0.037). eSCAR-positive patients were at higher risk of having SLE flares over follow-up (hazard ratio: 4.91; 95% CI 1.43–16.83; *p* = 0.0001). We identified inferoseptal myocardial scars by eSCAR in about one-fifth of SLE patients. Subclinical myocardial involvement was associated with glucocorticoid use and anti-dsDNA antibodies.

## 1. Introduction

Systemic lupus erythematosus (SLE) is a multi-systemic autoimmune and inflammatory disease burdened by increased mortality for cardiovascular disease (CVD) [1,2,3]. Across the spectrum of CVD involvement observed in patients with SLE, lupus cardiomyopathy has a high mortality [4,5]. Lupus cardiomyopathy is a poorly defined condition and is challenging to diagnose. However, its early identification among patients with SLE could reduce the risk of developing major cardiac arrhythmias, cardiogenic shock, acute coronary syndromes [6,7], or heart failure [8].

Currently, there are no shared guidelines regarding the characterization or treatment of cardiac involvement related to SLE. The best correlations with clinical data have been found with cardiovascular magnetic resonance (CMR) imaging, which revealed the presence of late gadolinium enhancement (LGE) in 30–40% of patients with SLE, mainly with non-ischemic, inflammatory distribution [9]. The prognostic role of myocardial scars (as detected by CMR-LGE imaging) has been confirmed by some longitudinal studies showing a significant association between the presence of myocardial scars and the risk of sudden cardiac death, arrhythmias, or heart failure both in patients with established ischemic heart disease and in those with primary cardiomyopathies or valvular heart diseases [10,11]. Thus, CMR imaging with the LGE technique is recognized as the non-invasive gold standard method for myocardial tissue characterization and myocardial scar detection [12,13]. However, the high costs, limited availability, long technical execution time, low patient compliance, and contraindications to the contrast agent limit the widespread application of CMR with the LGE technique for routine clinical use. Conversely, echocardiography, thanks to its widespread use, extreme portability of machinery and low costs, has now widely entered clinical practice as an essential diagnostic tool for routine use. Scar imaging echocardiography with ultrasound multi-pulse scheme (eSCAR) is a novel echocardiographic technique that is based on cancellation of the tissue signal through a sequence of pulses emitted by the probe (multipulse-scheme) in opposition of the phase or amplitude to each other. In addition, the eSCAR technique has been shown to have a good concordance rate with CMR-LGE in differentiating myocardial scarred tissue from the normal myocardial tissue in patients with recent acute myocardial infarction [14].

We reasoned that myocardial scar detection by the eSCAR technique could also contribute to risk stratification of SLE patients with preserved left ventricular function, who do not currently have effective prognostic stratification methods. To date, however, there are no studies that have investigated the presence of myocardial fibrosis as detected by eSCAR in patients with SLE. Hence, we designed the exploratory SCARLET (eSCAR in systemic Lupus ErythemaTosus) study with the primary aim to examine the feasibility of eSCAR for detecting myocardial scars in patients with SLE. Our secondary aim was to examine the association between the presence of myocardial scars and the clinical severity of SLE, defined as flares, glucocorticoid burden, and disease activity scores.

## 2. Materials and Methods

### 2.1. Study Population

Consecutive patients with a diagnosis of SLE, as per the American College of Rheumatology revised classification criteria, were screened at the Rheumatology outpatient service of the University Hospital of Verona between August 2019 and November 2020 (full inclusion and exclusion criteria of the study are described in Supplementary Data S1). Briefly, we included 27 patients with established SLE and stable drug treatment, while we excluded those with coexisting known diabetes mellitus and a prior history or symptoms of any CVD. A case-control sub-analysis included 32 subjects recruited for the study, named strain imaging in the evaluation of trastuzumab-related cardiotoxicity in patients with HER-2 positive breast cancer, who served as a control group. These subjects with newly diagnosed breast cancer, but who did not have any prior history of CVD, underwent a baseline echocardiographic examination before any cancer treatment was performed.

The local ethics committee approved the study protocol. All participants gave written informed consent for participation in the study.

### 2.2. Patient Involvement

This research was granted and approved by the association of patients Gruppo LES Italia Onlus. Patients who had SLE were not involved in setting the research question, study design, or outcome measures, but they were invited to comment on the results. Through this process, the involved SLE patients made highly value contributions by reporting more intelligible data in the final manuscript.

### 2.3. Study Outcomes

Participants were assessed at baseline for SLE and the traditional CVD risk factor profile. All participants underwent a trans-thoracic echocardiography exam (TTE) study completed with eSCAR evaluation and speckle tracking echocardiography (STE), and were then followed-up for one year. The primary outcome was the comparison of the presence of myocardial fibrosis by eSCAR between SLE patients and control subjects. The secondary outcome was the occurrence of SLE flares, which were assessed and classified according to the SELENA flare index [15]. The following clinically relevant events were also recorded during the follow-up (full described in Supplementary Data S2): all-causes of death, hospitalization, major cardiovascular events (MACE) [7], major arrhythmic events, heart failure (new-onset of dyspnea or myocardial dysfunction), peripheral artery disease, venous or arterial thromboembolism, cancer (excluding non-melanoma skin cancers), or infections requiring systemic antibiotic therapy.

### 2.4. Study Procedures

#### 2.4.1. Pulse-Cancellation Echocardiography

In order to perform eSCAR, the left ventricle contrast opacification (LVO) setting (power-modulation/pulse inversion harmonic imaging (transmit 1.6 MHz/receive 3.2 MHz)) was used for scar detection (eSCAR technique), without any contrast administration [16]. With this setting, the “linear” signals from normal myocardium were cancelled, while signals from abnormal myocardial tissue (fibrotic/disarrayed myocardium or calcified tissues) were enhanced as they had a “non-linear” response (similar to the non-linear acoustic behaviour of microbubbles). Starting from the 2D standard-setting, the “iscan” button, which automatically optimizes gain and time-gain compensation, was used once (set at 0 dB), after which the LVO setting was activated. The LVO setting was finely tuned to an intermediate mechanical index, between 0.40 and 0.47, and the general gain was set between 70% and 77%, depending on the individual subject echogenicity. This eSCAR setting exponentially enhanced the contrast between the scar and normal myocardium, allowing for the detection of myocardial fibrosis. A visual analysis of eSCAR images was used for assessment of the presence/absence and segmental distribution of myocardial scars by a blinded echocardiographer. A 17-segment model was used for assessing the myocardial segmental distribution of eSCAR signals.

#### 2.4.2. Speckle-Tracking Echocardiography

Speckle tracking echocardiography (STE) was also performed by a blinded echocardiographer using a dedicated commercially available Qlab 9 (cardiac motion quantification; Phillips Medical Systems) software package. Longitudinal strain for individual myocardial segments was measured from the apical 4-chamber, 2-chamber, and 3-chamber views (17-segment AHA/ASE model) [17]. In the end-diastole, automated border tracking was enabled before manual adjustment using a point and click approach to ensure that endocardial and epicardial borders were included in the region of interest. In the case of poor tracking, fine-tuning was performed manually after cine-loop playback and tracing were repeated, and it was adjusted until tracking was considered optimal by visual analysis. Individual myocardial segments that returned positive strain values and those with persistently poor tracking despite manual optimization were excluded from the analysis. The peak strain for the segment was defined as the peak negative value on the time strain curve for the entire cardiac cycle. The peak regional longitudinal strain was measured in 17 myocardial regions, and a weighted mean was used to derive the global longitudinal strain (GLS).

#### 2.4.3. CVD Risk Assessment

The following CVD risk factors were collected in all participants: age; sex; systolic blood pressure (SBP), diastolic blood pressure (DBP), and heart rate, which were measured at the end of echocardiographic evaluation in supine position; weight and height with the calculation of body mass index (BMI); waist circumference; plasma lipid profile including total cholesterol, triglycerides, low-density cholesterol, and high-density cholesterol; renal function parameters; and smoking status. We defined obesity as BMI ≥ 30 kg/m^2^. Dyslipidemia was defined as levels of total serum cholesterol ≥ 200 mg/dL and/or triglycerides ≥ 150 mg/dL, or the current use of any lipid-lowering drugs. Hypertension was defined as SBP ≥ 140 mmHg and/or DBP ≥ 90 mmHg, or the use of any anti-hypertensive agents. The glomerular filtration rate (eGFR) was estimated using the Chronic Kidney Disease Epidemiology Collaboration (CKD-EPI) equation [18], and CKD was defined as the eGFR < 60 mL/min per 1.73 m^2^.

#### 2.4.4. SLE Assessment

In all SLE patients, we collected data on the organ involvement, the medications used, and the daily dosage of glucocorticoid therapy. A rheumatologist systematically assessed disease activity and damage for each SLE patient and calculated both the Systemic Lupus Erythematosus Disease Activity Index 2000 (SLEDAI-2K) and the Systemic Lupus International Collaborating Clinics/American College of Rheumatology (SLICC) Damage Index (SDI).

#### 2.4.5. Laboratory Parameters

The following blood tests were performed in the local laboratory of our hospital: full blood count (FBC), complement fractions C3 and C4, erythrocyte sedimentation rate (ESR), C-reactive protein (CRP), and creatinine. The autoantibody status for each patient was also tested with commercially available assays, including measurements of anti-dsDNA antibodies (by indirect immunofluorescence and chemiluminescent immunoassay methods), anti-phospholipid antibodies (i.e., lupus anticoagulant, anti-beta2 glycoprotein I (GPI) IgG and IgM, and anti-cardiolipin IgG and IgM), and anti-extractable nuclear antigen (ENA) antibody panel.

### 2.5. Statistical Analysis

Given the exploratory design of the study, we did not perform a statistical power analysis. Frequency (categorical) variables were reported as absolute numbers and percentages. Continuous variables, except where otherwise defined, were reported as means and standard deviation. The associations of interest between categorical variables were analyzed with either the Chi-square test or the Fisher’s exact test where appropriate. The differences between the means for the eSCAR-positive and eSCAR-negative SLE patients were analyzed by either the unpaired Student’s *t* test or the Mann−Whitney U test where appropriate. We used the Kaplan−Meyer survival analysis and the log-rank test to compare the likelihood of remaining SLE flare-free during the follow-up period in eSCAR-positive vs. eSCAR-negative patients. Statistical significance was considered for a value of *p* < 0.05. All statistical analyses were performed using the IBM SPSS Statistics for Windows version 20 software (IBM Corp, Armonk, NY, USA) and the GraphPad Prism version 7 for Windows (GraphPad Software, San Diego, CA, USA).

## 3. Results

### 3.1. Baseline Characteristics of SLE Patients

We screened 60 consecutive SLE patients. After applying the inclusion and exclusion criteria, we enrolled 27 patients with established SLE for the analysis (study flow-chart illustrated in Supplementary Figure S1). We also included 32 control subjects matched for age, sex, and traditional CVD risk factors for the case-control study. By study design, none of these participants had established diabetes or pre-existing history of CVD.

Most patients with SLE were affected by long-standing disease, with an age of onset being about 29 years and an average time since disease onset of 14 years. The most common SLE-related symptoms were arthritis (74%), mucocutaneous manifestations (59%), and lupus nephritis (44%). Nineteen percent of these patients fulfilled criteria for antiphospholipid antibody syndrome, mostly obstetric type. Disease-associated damage and disease activity were low overall, with 7/27 (26%) patients having a SLEDAI-2K of zero; 56% of patients had decreased serum complement C3 or C4 fragments, and anti-dsDNA antibodies were detected in 74% of cases. Most SLE patients were receiving combinations of hydroxychloroquine, immunosuppressants, and glucocorticoids (mean prednisone dose of 3.8 mg daily). There were no patients taking NSAIDs chronically, and only 4/27 were taking oral NSAIDs as needed.

### 3.2. Detection of Myocardial Scars by eSCAR in SLE Patients

**Figure 1:**
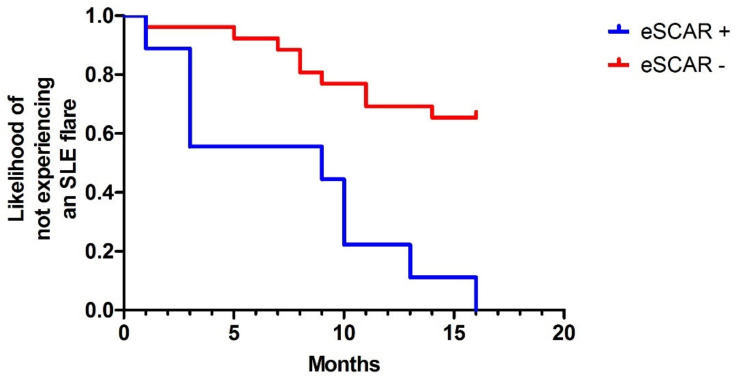
On the left: myocardial fibrosis in five patients with SLE, as described by a 17-segment “bull’s eye” scheme. Yellow segments show the localization of the echocardiographic scar (eSCAR) sign as detected by pulse cancellation imaging. On the right: the presence of myocardial fibrosis in the interventricular septum (i.e., eSCAR-positive patient) is shown in panel (**A**). The absence of myocardial fibrosis (eSCAR-negative patient) is shown in panel (**B**).

As shown in Table 1, traditional CVD risk factors did not significantly differ between SLE patients and controls. Myocardial scars as detected by the eSCAR technique were found in 5/27 (19%) patients with SLE and in none of the control group (Table 2). The myocardial infero-septal segments were affected in all these eSCAR-positive patients, and only in one case the inferior myocardial wall was also affected (Figure 1). GLS in most myocardial segments was significantly decreased in SLE patients compared with the controls, except for the apical region. All of the participants had a preserved LV function. Compared with the controls, the LV end-diastolic (LV-EDV) and end-systolic volumes (LV-ESV) were higher in SLE patients, while the LV ejection fraction (LV-EF) was lower, although within the normal range. The s’ tricuspid wave velocity was lower in SLE patients than in the controls. Conversely, there were no significant differences between the two groups in terms of LV mass, left atrial volume, and diastolic function indexes.

### 3.3. Association of Myocardial Scar by eSCAR with Impaired Myocardial Strain in SLE Patients

We compared the echocardiographic data between SLE patients with myocardial scars (termed eSCAR-positive, *n* = 5) and those without myocardial scars (termed eSCAR-negative, *n* = 22). The myocardial strain values were significantly lower in the eSCAR-positive group, mainly in the basal and infero-septal segments (Table 2 and Supplementary Figure S2). Other echocardiographic parameters were similar between the two groups of patients.

### 3.4. Association of Myocardial Scars by eSCAR with Glucocorticoids and Anti-dsDNA

There were no significant differences in terms of the distribution of CVD risk factors, clinical manifestations, disease duration, SDI, or SLEDAI-2K between the two groups (Table 3). Conversely, the serum levels of ANA and anti-dsDNA antibodies were significantly higher in eSCAR-positive patients compared with eSCAR-negative patients. eSCAR-positive patients were taking a numerically higher cumulative glucocorticoid dose, as well as a higher current daily glucocorticoid dose compared with the eSCAR-positive patients, although these differences were not statistically significant (Table 3); however, we also found a significant inverse association between LV mass and cumulative prednisone dose (Supplementary Figure S3). Full blood counts, haemoglobin, renal function, inflammatory markers, and complement C3 and C4 levels did not significantly differ between the two groups of patients (Table 3).

### 3.5. Association of Myocardial Scars by eSCAR with SLE Flares

At the end of the follow-up period, 18 episodes of SLE flares were recorded in 11/27 (41%) patients. Four patients had more than one flare. In 6/11 (55%) patients, flares were classified as mild/moderate, while they were severe in 5/11 patients; no flares required hospitalization.

**Figure 2:**
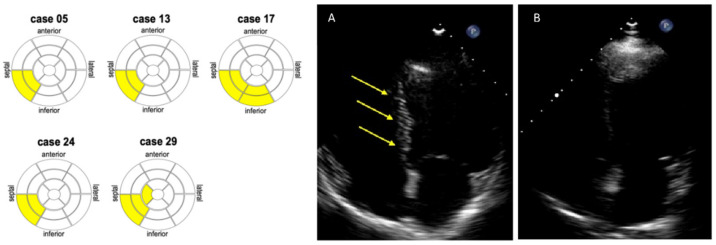
Survival curves for the status of SLE patients maintaining flare-free status over the follow-up period of the study. Hazard ratio for eSCAR-positive vs. eSCAR-negative patients: 4.91; 95% CI 1.43–16.83; *p* = 0.0001 (by the log-rank test).

We recorded nine flares in 5/5 participants in the eSCAR-positive group compared with nine flares in 6/22 eSCAR-negative SLE patients (100% vs. 27%, *p* = 0.006). All eSCAR-positive patients had at least one flare and 3/5 more than one flare. Compared with the eSCAR-negative patients, eSCAR-positive patients were significantly less likely to maintaining their flare-free status during the 1-year follow-up period (Figure 2), as half of them had an SLE flare within 9 months following the baseline visit. Conversely, the rates of other clinically relevant events were comparable between the two groups of patients (Supplementary Table S2 and Supplementary Data S3).

## 4. Discussion

This pilot observational study provided preliminary data supporting that the eSCAR technique is feasible in patients with SLE and that it can effectively detect subclinical myocardial involvement in this patient population. The eSCAR technique is exquisitely appealing to rheumatologists, who are familiar to the use of ultrasounds for musculoskeletal imaging, and here it was fast and easy to perform in all study participants.

eSCAR was a specific finding in SLE patients as it was not detected in the control group. Notably, in all eSCAR-positive patients (19% of total), the infero-septal myocardial segment was affected, thus supporting a common pattern in terms of the localization of myocardial fibrosis. These eSCAR-positive patients also had standard TTE values within the normal ranges, thereby suggesting subclinical myocardial involvement. Significant differences in the indexed volumes and standard LV function parameters were also noticed in SLE patients compared with the controls; however, they resulted within the normal limits in both groups. Indeed, our results are consistent with the low pre-test risk of structural heart disease, as no participant had a prior history or any symptoms attributable to CVD.

Interestingly, the subclinical myocardial involvement we observed in SLE patients was also confirmed by STE findings, showing impaired GLS values in patients with SLE compared with the controls, except for the apical region. STE is an advanced echocardiographic method that is able to assess and quantify myocardial active deformation, and it has been proven to be a more sensitive technique compared with conventional TTE for the early diagnosis of subclinical lupus cardiomyopathy [19,20]. We found abnormal GLS values in most myocardial segments, but they were more significantly altered in the infero-septal segments, which are the same areas where we detected myocardial scars through eSCAR. This finding supports the notion that myocardial tissue damage and scarring might occur subclinically in SLE patients, thereby inducing early abnormalities in myocardial active deformation as assessed by STE, in the presence of otherwise normal conventional TTE examinations.

Early myocardial fibrosis could also have some clinical prognostic implications in SLE patients over time. Indeed, we found that eSCAR-positive patients were more likely to have at least one or more SLE flares during the 1-year follow-up of the study compared with eSCAR-negative patients. This result suggests that SLE patients with myocardial involvement by eSCAR could also have had a more active and aggressive disease, requiring a higher dose of glucocorticoids in order to keep it in remission. In support of this hypothesis, we found that compared with the eSCAR-negative group, eSCAR-positive patients had a higher cumulative glucocorticoid exposure that was significantly correlated with LV mass, as well as a higher daily dose of oral prednisone. Furthermore, eSCAR-positive patients had significantly higher levels of circulating anti-dsDNA antibodies, which have been associated with an increased risk of SLE flare [21]. Jointly, all these data support the slightly higher disease activity as assessed by the SLEDAI in the eSCAR-positive group—a score that currently does not consider the presence of cardiac involvement [22].

eSCAR in SLE patients might indicate a predominantly non-ischemic, inflammatory fibrosis. Small studies using LGE-CMR have recently proven non-ischemic scarring to be more common than ischemic scars in asymptomatic SLE patients with no prior history of cardiac disease. Puntman et al. reported the presence of myocardial LGE in almost two thirds of SLE patients, predominantly in the inferolateral-inferior and infero-septal basal-mid segments, with an intramyocardial or epicardial distribution [23]. Winau et al. found LGE-CMR in 30% of SLE subjects (6.7% of which were the ischemic type) [9]. Burkard et al. found LGE-CMR in about 30% (9/30) of SLE patients, all with a myocardial non-ischemic distribution [24]. The LGE-CMR appearance in these studies closely resembles the typical pattern observed in chronic myocarditis or idiopathic-dilated cardiomyopathy, often presenting with a septal intramyocardial stria of infero-lateral epicardial scarring [25]. It has been hypothesized that in asymptomatic SLE patients, these LGE-CMR findings may represent the result of an indolent course of mild perimyocarditis. Collectively, our findings using the eSCAR technique are in keeping with the aforementioned observations based on the use of LGE-CMR.

*Study Limitations and Strengths*

First, an obvious limitation of our study is its relatively small sample size, which can be accepted as per its pilot design. Second, SLE patients with severe organ involvement (in particular renal and vascular) were under-represented. Therefore, we might have underestimated the prevalence of lupus myocarditis, which is generally associated with more severe disease activity patterns. On the other hand, it is remarkable to have found areas of myocardial fibrosis in about one of five patients with clinically quiescent disease. Third, the follow-up length of our study was limited to a period of 1 year, which is too short to assess whether there was a higher incidence of CVD mortality and morbidity in eSCAR-positive patients than in eSCAR-negative patients. Fourth, the validation of the eSCAR technique through CMR is advised, but it is currently underway. However, the eSCAR technique has already been validated with CMR-LGE in patients with recent myocardial infarction [14]. Finally, we did not assess myocardial injury biomarkers that could indicate the presence of subclinical cardiac damage in those patients with fibrosis.

## 5. Conclusions

The eSCAR technique efficiently detected subclinical myocardial damage in SLE patients and suggested its prognostic role in predicting SLE flares. Such new imaging technique could be routinely used for the cardiac surveillance and management of patients with SLE, as it has been proven to be easy, cheap, fast, and well tolerated.

## Figures and Tables

**Table 1:** Cardiovascular risk profile and echocardiographic features in SLE patients and control subjects.

	SLE Patients (*n* = 27)	Controls (*n* = 32)	*p*-Value
**Cardiovascular risk factors**			
Age, years	45 ± 11	46 ± 7	0.797
Male sex, *n* (%)	3 (11)	0 (0)	0.090
BMI, kg/m^2^	23 ± 3	23 ± 4	0.999
Current smokers, *n* (%)	10 (37)	8 (25)	0.399
Hypertension, *n* (%)	8 (30)	3 (9)	0.091
Hypercholesterolemia, *n* (%)	4 (15)	6 (19)	0.728
**Standard echocardiography**			
LV EDV index, mL/m^2^	53.8 ± 11	49.1 ± 6.9	0.04
LV ESV index, mL/m^2^	20.9 ± 5.2	17.9 ± 3.7	0.01
LV EF, %	61.2 ± 4.2	63.7 ± 2.9	0.009
LV mass index, g/m^2^	64 ± 14.7	65 ± 17.6	0.87
LAVI, mL/m^2^	22.8 ± 6.9	24 ± 6.3	0.49
E velocity (cm/s)	74.3 ± 21.7	77.9 ± 17.8	0.47
A velocity (cm/s)	60 ± 18.6	66.6 ± 17.6	0.16
Deceleration time, ms	183.8 ± 74.5	182.5 ± 60.7	0.94
E/A ratio	1.3 ± 0.6	1.2 ± 0.4	0.72
E/e’ ratio	6.9 ± 2.5	6.7 ± 2.1	0.85
TRPG, mmHg	17.5 ± 4.1	19.4 ± 4.3	0.29
TAPSE, mmHg	24 ± 7.2	24.3 ± 2.7	0.82
s’ tricuspidal velocity, cm/s	10.3 ± 5.1	13.2 ± 1.7	0.01
**Speckle tracking echocardiography**			
GLS global (%)	−21 ± 2	−23.9 ± 1.8	<0.0001
GLS 4-chamber (%)	−21.5 ± 2.7	−22.8 ± 1.9	0.03
GLS 2-chamber (%)	−21.6 ± 2.4	−22.8 ± 2.1	0.04
GLS 3-chamber (%)	−20.9 ± 2.6	−22.5 ± 2.4	0.01
GLS base (%)	−19 ± 2.6	−22.8 ± 2.9	<0.0001
GLS mid (%)	−19.5 ± 2	−23.5 ± 3.4	<0.0001
GLS apex (%)	−25.1 ± 3	−25.5 ± 3.3	0.6
GLS anterior (%)	−21.9 ± 2.4	−23.8 ± 4.3	0.03
GLS antero-septal (%)	−22.6 ± 3.2	−25.8 ± 3.6	0.001
GLS infero-septal (%)	−20.9 ± 2.5	−23.5 ± 2.8	<0.0001
GLS inferior (%)	−21.2 ± 2.4	−25 ± 3.5	<0.0001
GLS infero-lateral (%)	−20.3 ± 2.6	−22.6 ± 2.7	0.001
GLS antero-lateral (%)	−21.4 ± 2.7	−23.5 ± 2.7	0.004
**Myocardial fibrosis (eSCAR)**			
eSCAR, *n* (%)	5 (19)	0 (0)	0.01
eSCAR anterior, *n* (%)	0 (0)	0 (0)	ND
eSCAR antero-septal, *n* (%)	0 (0)	0 (0)	ND
eSCAR infero-septal, *n* (%)	5 (19)	0 (0)	0.01
eSCAR inferior, *n* (%)	1 (4)	0 (0)	0.29
eSCAR infero-lateral, *n* (%)	0 (0)	0 (0)	ND
eSCAR antero-lateral, *n* (%)	0 (0)	0 (0)	ND
eSCAR, *n* (%)	5 (19)	0 (0)	0.01
eSCAR anterior, *n* (%)	0 (0)	0 (0)	ND
eSCAR antero-septal, *n* (%)	0 (0)	0 (0)	ND
eSCAR infero-septal, *n* (%)	5 (19)	0 (0)	0.01

Data reported as numbers (percentages) or means ± SD. BMI, body mass index; EDV, end-diastolic volume; EF, ejection fraction; ESV, end-systolic volume; GLS, global longitudinal strain; LAVI, left atrial volume index; LV, left ventricular; ND, not determined; SLE, systemic lupus erhytematosus; TAPSE, tricuspid annular plane systolic excursion; TRPG, tricuspid regurgitation peak gradient.

**Table 2:** Standard echocardiographic and myocardial strain data in patients with SLE stratified by positivity of eSCAR imaging technique.

	eSCAR-Positive (*n* = 5)	eSCAR-Negative (*n* = 22)	*p*-Value
** Standard echocardiography **			
LV EDV index, mL/m^2^	56.7 ± 18.5	53.3 ± 9.3	0.53
LV ESV index, mL/m^2^	22.4 ± 8	20.7 ± 4.7	0.53
LV EF, %	60.7 ± 3.2	61.3 ± 4.4	0.76
LV mass index, g/m^2^	67.7 ± 20.7	63.3 ± 13.6	0.55
LAVI, mL/m^2^	19.8 ± 7.7	23.5 ± 6.7	0.28
E velocity (cm/s)	76.2 ± 15.1	73.8 ± 23.1	0.82
A velocity (cm/s)	56.5 ± 20.6	60.8 ± 18.6	0.64
Deceleration time, ms	225.2 ± 30.8	175.1 ± 78.4	0.17
E/A ratio	1.5 ± 0.6	1.2 ± 0.5	0.37
E/e’ ratio	7.8 ± 3.7	6.7 ± 2.2	0.40
TRPG, mmHg	22 ± 2.4	25.2 ± 7.6	0.46
TAPSE, mmHg	12 ± 1.8	9.9 ± 5.5	0.47
s’ tricuspidal velocity, cm/s	56.7 ± 18.5	53.3 ± 9.3	0.53
** Speckle tracking echocardiography **			
GLS global (%)	−18.4 ± 1.5	−21.6 ± 1.7	0.001
GLS 4-chamber (%)	−18.2 ± 2.2	−22.2 ± 2.3	0.002
GLS 2-chamber (%)	−18.9 ± 1.9	−22.2 ± 2.1	0.003
GLS 3-chamber (%)	−19.8 ± 3.6	−21.1 ± 2.4	0.31
GLS base (%)	−15.7 ± 2.4	−19.7 ± 2.2	0.001
GLS mid (%)	−17.3 ± 1.4	−20 ± 1.9	0.005
GLS apex (%)	−23.1 ± 1.1	−25.5 ± 3.1	0.1
GLS anterior (%)	−18.8 ± 1.9	−22.5 ± 2	0.001
GLS antero-septal (%)	−20.7 ± 2.1	−23.1 ± 3.3	0.13
GLS infero-septal (%)	−17.3 ± 2.1	−21.7 ± 1.9	<0.0001
GLS inferior (%)	−18.5 ± 2.1	−21.7 ± 2.2	0.006
GLS infero-lateral (%)	−18.3 ± 3.3	−20.7 ± 2.4	0.05
GLS antero-lateral (%)	−18.8 ± 2.7	−21.9 ± 2.4	0.01

EDV, end-diastolic volume; EF, ejection fraction; ESV, end-systolic volume; GLS, global longitudinal strain; LAVI, left atrial volume index; LV, left ventricular; SLE, systemic lupus erhytematosus; TAPSE, tricuspid annular plane systolic excursion; TRPG, tricuspid regurgitation peak gradient.

**Table 3:** Main clinical and biochemical characteristics in eSCAR-positive and eSCAR-negative patients with SLE.

	eSCAR-Positive (*n* = 5)	eSCAR-Negative (*n* = 22)	*p*-Value
** Cardiovascular risk factors **			
Age, years	41 (33, 50)	45 (39, 54)	0.151
Female sex, *n* (%)	4 (80)	20 (91)	0.999
Obesity, *n* (%)	0 (0)	1 (5)	0.999
Smoking (ever), *n* (%)	3 (60)	7 (32)	0.326
Hypertension, *n* (%)	2 (40)	6 (27)	0.616
Dyslipidemia, *n* (%)	0 (0)	4 (18)	0.561
** SLE characteristics **			
Disease duration, years	11 (3, 24)	13 (8, 21)	0.901
Age at diagnosis, years	23 (17, 45)	29 (23, 34)	0.289
SLEDAI score	5 (2.5, 10.5)	2.0 (2.0, 6.0)	0.161
SDI score	0.5 (0.0, 1.8)	1.0 (0.0, 2.0)	0.973
Arthritis, *n* (%)	3 (60)	15 (68)	0.999
Neuropsychiatric manifestations, *n* (%)	0 (0)	3 (14)	0.999
Lupus nephritis, *n* (%)	1 (20)	11 (50)	0.342
Mucocutaneous manifestations, *n* (%)	3 (60)	12 (55)	0.999
Cytopenia, *n* (%)	3 (60)	8 (36)	0.370
Antiphospholipid syndrome, *n* (%)	1 (20)	5 (23)	0.999
Serositis, *n* (%)	0 (0)	3 (14)	0.999
** Laboratory data **			
Hemoglobin, g/dL	12.9 (12.6, 13.5)	13.0 (12.0, 14.1)	0.731
Creatinine, mg/dL	0.81 (0.55, 1.51)	0.67 (0.60, 0.74)	0.377
eGFR, mL/min per 1.73 m^2^	103 (87, 121)	100 (69, 161)	0.454
ESR, mm/h	15 (6, 28)	15 (6, 21)	0.433
CRP, mg/L	1.5 (0.2, 2.8)	2.0 (1.0, 4.0)	0.271
Complement C3, g/L	71 (60, 91)	87 (70, 99)	0.142
Complement C4, g/L	12 (6, 30)	15 (8, 18)	0.901
** Autoantibodies **			
Antinuclear Ab (≥1:80) IIF, *n* (%)	5 (100)	22 (100)	0.999
Antinuclear Ab titer, dilution	1:1280 (1:640, 1:1280)	1:320 (1:160, 1:640)	0.019
Anti-dsDNA Ab IIF, *n* (%)	4 (80)	15 (68)	0.540
Anti-dsDNA Ab titer, IU/mL	127 (55, 286)	21 (0, 54)	0.037
Anti-Smith Ab, *n* (%)	2 (40)	4 (18)	0.303
Anti-Ro/SSA Ab, *n* (%)	2 (40)	9 (41)	0.999
Anti-La/SSB Ab, *n* (%)	0 (0)	6 (27)	0.555
Anti-U1RNP Ab, *n* (%)	2 (40)	4 (18)	0.303
Antiphospholipid Ab, *n* (%)	2 (40)	9 (41)	0.999
** SLE medications **			
Glucocorticoids, *n* (%)	4 (80)	12 (55)	0.618
Current prednisone dose, mg day	11 (1, 27)	3 (0, 5)	0.067
Cumulative prednisone dose, g	36 (6, 61)	19 (5, 26)	0.212
Hydroxychloroquine, *n* (%)	3 (60)	19 (86)	0.221
Methotrexate, *n* (%)	1 (20)	3 (14)	0.999
Azathioprine, *n* (%)	0 (0)	2 (9)	0.999
Mycophenolate, *n* (%)	1 (20)	9 (41)	0.621
Belimumab, *n* (%)	2 (40)	5 (23)	0.580
Prior cyclophosphamide, *n* (%)	0 (0)	3 (14)	0.999
Prior rituximab, *n* (%)	2 (40)	2 (9)	0.144

Continuous variables are reported as medians (25th, 75th percentile). Ab, antibodies; BMI, body mass index; CRP, C-reactive protein; eGFR, estimated glomerular filtration rate; ESR, erythrocyte sedimentation rate; IIF, indirect immunofluorescence; SDI, SLICC damage index; SLE, systemic lupus erhytematosus; SLEDAI, Systemic Lupus Erythematosus Disease Activity Index 2000.

## Data Availability

The data presented in this study are available upon request from the corresponding author.

## Supplementary Materials

The following supporting information can be downloaded at: https://www.mdpi.com/article/10.3390/jcm11164788/s1, Figure S1: The SCARLET study flow-chart; Figure S2: Difference in GLS of the infero-septal wall in the presence or absence of myocardial scars as detected by eSCAR; Figure S3: Associations of relative wall thickness (RWT) or left ventricular mass index (LVMI) (vertical axis) with cumulative prednisone dose expressed in grams (horizontal axis); Table S1: Adverse clinical outcomes and disease flares in eSCAR-positive and eSCAR-negative patients with SLE over the 1-year follow-up period; Data S1: Inclusion and exclusion criteria; Data S2: Events of clinical relevance recorded during follow-up.; Data S3: Description of clinical events.

## References

[1] Bengtsson A.A., Rylander L., Hagmar L., Nived O., Sturfelt G. (2002). Risk factors for developing systemic lupus erythematosus: A case-control study in southern Sweden. Rheumatology.

[2] Palmieri V., Migliaresi P., Orefice M., Lupo T., Di Minno M., Valentini G., Celentano A. (2009). High prevalence of subclinical cardiovascular abnormalities in patients with systemic lupus erythematosus in spite of a very low clinical damage index. Nutr. Metab. Cardiovasc. Dis..

[3] Manzi S., Meilahn E.N., Rairie J.E., Conte C.G., Medsger T.A., Jansen-McWilliams L., D’Agostino R.B., Kuller L.H. (1997). Age-specific Incidence Rates of Myocardial Infarction and Angina in Women with Systemic Lupus Erythematosus: Comparison with the Framingham Study. Am. J. Epidemiol..

[4] Apte M., McGwin J.G., Vila L.M., Kaslow R.A., Alarcon G.S., Reveille J.D., LUMINA Study Group (2007). Associated factors and impact of myocarditis in patients with SLE from LUMINA, a multiethnic US cohort. Rheumatology.

[5] Jacobsen S., Petersen J., Ullman S., Junker P., Voss A., Rasmussen J.M., Tarp U., Poulsen L.H., Hansen G.V.O., Skaarup B. (1998). A multicentre study of 513 Danish patients with systemic lupus erythematosus. II. Disease mortality and clinical factors of prognostic value. Clin. Rheumatol..

[6] Tanwani J., Tselios K., Gladman D.D., Su J., Urowitz M.B. (2018). Lupus myocarditis: A single center experience and a comparative analysis of observational cohort studies. Lupus.

[7] Hicks K.A., Mahaffey K.W., Mehran R., Nissen S.E., Wiviott S.D., Dunn W., Solomon S.D., Marler J.R., Teerlink J.R., Farb A. (2018). 2017 Cardiovascular and Stroke Endpoint Definitions for Clinical Trials. Circulation.

[8] Comarmond C., Cacoub P. (2017). Myocarditis in auto-immune or auto-inflammatory diseases. Autoimmun. Rev..

[9] Winau L., Baydes R.H., Braner A., Drott U., Burkhardt H., Sangle S., D’Cruz D.P., Carr-White G., Marber M., Schnoes K. (2018). High-sensitive troponin is associated with subclinical imaging biosignature of inflammatory cardiovascular involvement in systemic lupus erythematosus. Ann. Rheum. Dis..

[10] Kwong R.Y., Chan A.K., Brown K.A., Chan C.W., Reynolds H.G., Tsang S., Davis R.B. (2006). Impact of Unrecognized Myocardial Scar Detected by Cardiac Magnetic Resonance Imaging on Event-Free Survival in Patients Presenting with Signs or Symptoms of Coronary Artery Disease. Circulation.

[11] Gulati A., Jabbour A., Ismail T.F., Guha K., Khwaja J., Raza S., Morarji K., Brown T.D.H., Ismail N.A., Dweck M.R. (2013). Association of Fibrosis with Mortality and Sudden Cardiac Death in Patients with Nonischemic Dilated Cardiomyopathy. JAMA.

[12] Wu E., Judd R.M., Vargas J.D., Klocke F.J., Bonow R.O., Kim R.J. (2001). Visualisation of presence, location, and transmural extent of healed Q-wave and non-Q-wave myocardial infarction. Lancet.

[13] Wagner A., Mahrholdt H., Holly T.A., Elliott M.D., Regenfus M., Parker M., Klocke F.J., Bonow R.O., Kim R.J., Judd R.M. (2003). Contrast-enhanced MRI and routine single photon emission computed tomography (SPECT) perfusion imaging for detection of subendocardial myocardial infarcts: An imaging study. Lancet.

[14] Gaibazzi N., Bianconcini M., Marziliano N., Parrini I., Conte M.R., Siniscalchi C., Faden G., Faggiano P., Pigazzani F., Grassi F. (2016). Scar Detection by Pulse-Cancellation Echocardiography. JACC: Cardiovasc. Imaging.

[15] Petri M., Buyon J., Kim M. (1999). Classification and definition of major flares in SLE clinical trials. Lupus.

[16] Gaibazzi N., Tuttolomondo D., Guaricci A.I., Di Giannuario G. (2021). Pulse-Cancellation Echocardiography for Clinical Evaluation of Myocardial Scar Burden. Curr. Cardiol. Rep..

[17] Mor-Avi V., Lang R.M., Badano L.P., Belohlavek M., Cardim N.M., Derumeaux G., Galderisi M., Marwick T., Nagueh S.F., Sengupta P.P. (2011). Current and Evolving Echocardiographic Techniques for the Quantitative Evaluation of Cardiac Mechanics: ASE/EAE Consensus Statement on Methodology and Indications endorsed by the Japanese Society of Echocardiography. J. Am. Soc. Echocardiogr..

[18] Levey A.S., Stevens L.A., Schmid C.H., Zhang Y.L., Castro A.F., Feldman H.I., Kusek J.W., Eggers P., Van Lente F., Greene T. (2009). A New Equation to Estimate Glomerular Filtration Rate. Ann. Intern. Med..

[19] Du Toit R., Herbst P.G., van Rensburg A., Snyman H.W., Reuter H., Doubell A.F. (2017). Speckle tracking echocardiography in acute lupus myocarditis: Comparison to conventional echocardiography. Echo Res. Pract..

[20] Farag S.I., Bastawisy R.B., Hamouda M.A., Hassib W.A., Wahdan H.A. (2020). Value of speckle tracking echocardiography for early detection of left ventricular dysfunction in patients with systemic lupus erythematosus. J. Cardiovasc. Echogr..

[21] Zen M., Bassi N., Nalotto L., Canova M., Bettio S., Gatto M., Ghirardello A., Iaccarino L., Punzi L., Doria A. (2012). Disease activity patterns in a monocentric cohort of SLE patients: A seven-year follow-up study. Clin. Exp. Rheumatol..

[22] Gladman D.D., Ibañez D., Urowitz M.B. (2002). Systemic lupus erythematosus disease activity index 2000. J. Rheumatol..

[23] Puntmann V.O., D’Cruz D., Smith Z., Pastor A., Choong P., Voigt T., Carr-White G., Sangle S., Schaeffter T., Nagel E. (2013). Native Myocardial T1 Mapping by Cardiovascular Magnetic Resonance Imaging in Subclinical Cardiomyopathy in Patients with Systemic Lupus Erythematosus. Circ. Cardiovasc. Imaging.

[24] Burkard T., Trendelenburg M., Daikeler T., Hess C., Bremerich J., Haaf P., Buser P., Zellweger M.J. (2018). The heart in systemic lupus erythematosus—A comprehensive approach by cardiovascular magnetic resonance tomography. PLoS ONE.

[25] Assomull R.G., Prasad S.K., Lyne J., Smith G., Burman E.D., Khan M., Sheppard M.N., Poole-Wilson P.A., Pennell D.J. (2006). Cardiovascular Magnetic Resonance, Fibrosis, and Prognosis in Dilated Cardiomyopathy. J. Am. Coll. Cardiol..

