# Supplementary material for: Subclinical Myocardial Fibrosis in Systemic Lupus Erythematosus as Assessed by Pulse-Cancellation Echocardiography: A Pilot Study
Source: J Clin Med. 2022 Aug 16;11(16):4788. doi: 10.3390/jcm11164788 (PMC9410017; doi:10.3390/jcm11164788)
Supplement: Supplementary file 1 [file jcm-11-04788-s001.zip › jcm-1807707-supplementary.pdf]

SCARLET Study | Supplementary data files

Supplementary Table S1. Adverse clinical outcomes and disease flares in eSCAR-positive and eSCAR-negative patients with SLE over the 1-year follow-up period.

	**eSCAR-positive (n = 5)**	**eSCAR-negative (n = 22)**	**P-value**
Major CVD events, n(%)	0 (0)	1 (5)	0.999
Malignancy, n(%)	0 (0)	1 (5)	0.999
Infections, n(%)	1 (20)	5 (23)	0.999
SLE flares, n(%)	5 (100)	6 (27)	0.006
Death, n(%)	0 (0)	0 (0)	0.999

CVD, cardiovascular disease; SLE, systemic lupus erhytematosus.

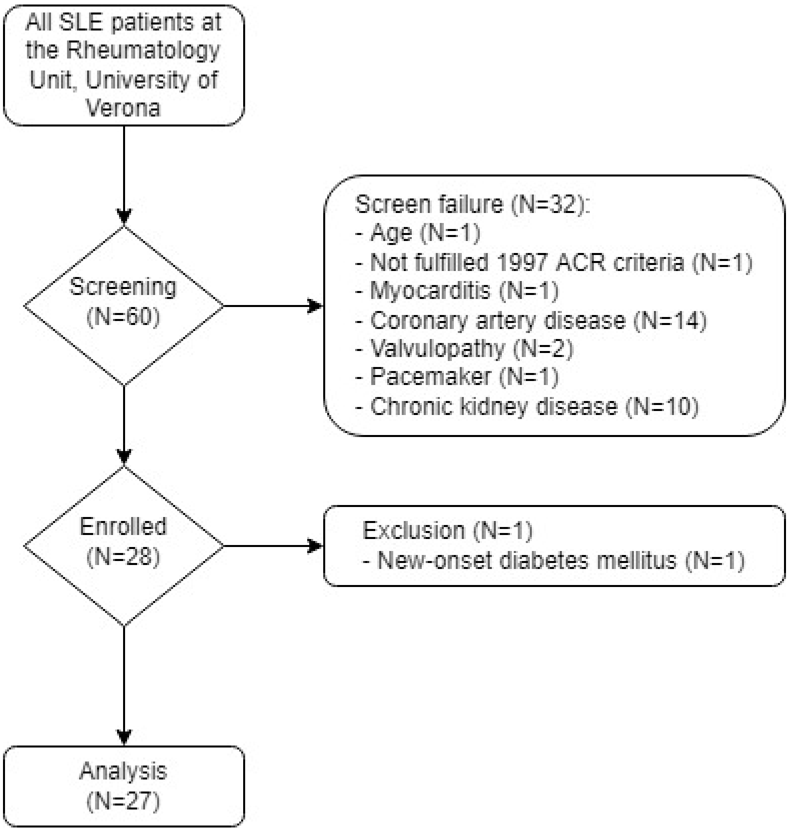

Supplementary Figure S1. The SCARLET study flow-chart.

ACR, American College of Rheumatology.

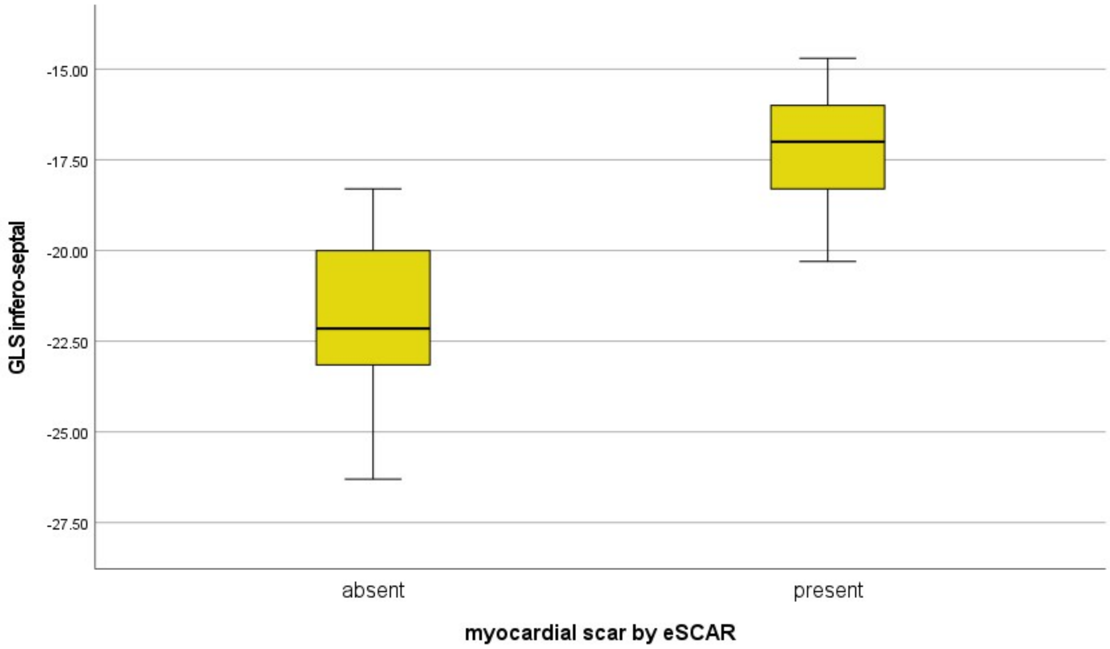

Supplementary Figure S2. Difference in GLS of the infero-septal wall in the presence or absence of myocardial scars as detected by eSCAR.

Data are shown as medians and interquartile ranges for each subgroup.

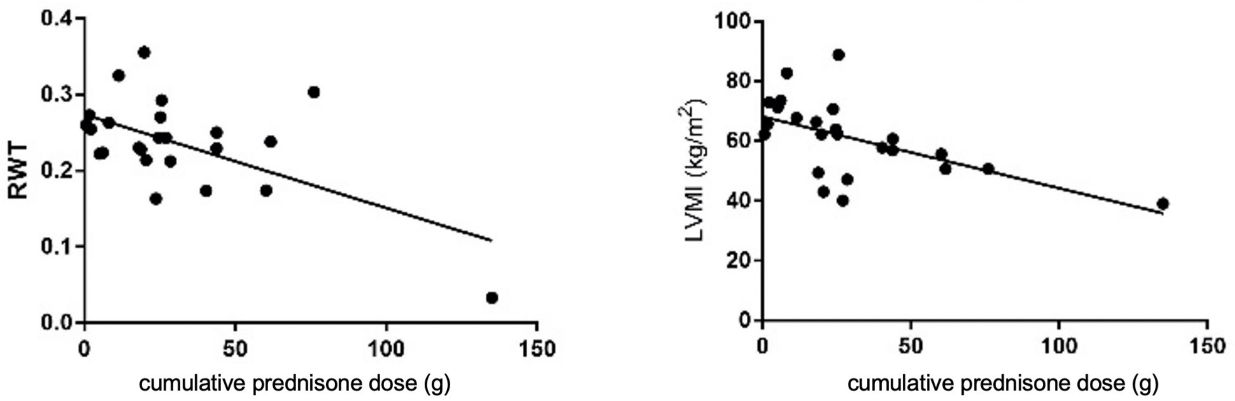

Supplementary Figure S3. Associations of relative wall thickness (RWT) or left ventricular mass index (LVMI) (vertical axis) with cumulative prednisone dose expressed in grams (horizontal axis). Pearson's r and p-values were: r = -0.587, p=0.003 for RWT; and r = -0.565, p=0.004 for LVMI, respectively.

Supplementary Data S1. Inclusion and exclusion criteria

**Inclusion criteria**
- Age between 18 and 65 years<br>- Diagnosis of SLE as per the 1997 American College of Rheumatology revised classification criteria<br>- Signed informed consent
**Exclusion criteria**
- Previous or current overt myocarditis/pericarditis<br>- Systolic or diastolic left ventricular dysfunction on echocardiography<br>- Coronary artery disease, previous myocardial infarction or previous percutaneous or surgical coronary revascularization procedures<br>- Severe heart valve diseases or previous heart valve surgery<br>- Patients with pacemaker or implantable cardioverter-defibrillator<br>- Life expectancy of less than 2 years for any cause<br>- Congenital heart diseases<br>- End-stage renal failure or kidney transplantation<br>- Type 2 or type 1 diabetes mellitus

Supplementary Data S2. Events of clinical relevance recorded during follow-up.

1. All-cause death;
2. Hospitalisations for CVD and non-CVD events;
3. Major cardiovascular events [14]:
    a. Stroke and transient ischemic attack;
    b. Acute coronary syndrome (acute myocardial infarction, unstable angina);
    c. Revascularisation (aorto-coronary bypass, percutaneous transcatheter angioplasty);
4. Cardiac arrhythmic events (atrial fibrillation, atrial flutter, supraventricular tachycardia; ventricular tachycardia or fibrillation, sudden cardiac death, appropriate shock; atrioventricular block);
5. Heart failure (new-onset of dyspnea or myocardial dysfunction);
6. Peripheral artery disease (claudication intermittent or arterial vascular ulcers);
7. Venous or arterial thromboembolism;
8. Cancer (excluding non-melanoma skin cancers);
9. Infection requiring systemic antibiotic therapy.
10. SLE flare according to the SELENA trial definitions.

Supplementary Data S3. Description of clinical events

Thirty-one clinical events were reported in 15/27 (56%) patients. These events were as follows: ten infections requiring antibiotic therapy, two CVD events, and one malignancy. There were no deaths. In the eSCAR-positive group, there were two infections in the same patient (pauci-symptomatic Severe Acute Respiratory Syndrome (SARS)-CoronaVirus2 (CoV2) infection and bacterial pneumonia). In the eSCAR-negative group, there were five infections (one episode of recurrent nail infection with hospitalization for onychectomy; one episode of upper respiratory tract infection; one lower respiratory tract infection; one relapsed erysipelas, and one SARS-CoV2 infection), one cancer (renal cell carcinoma), and one cardiovascular event (arterial ulcer).
